# Children with SARS-CoV-2 infection during the novel coronaviral disease (COVID-19) outbreak in Iran: an alarming concern for severity and mortality of the disease

**DOI:** 10.1186/s12879-022-07200-0

**Published:** 2022-04-15

**Authors:** Setareh Mamishi, Babak Pourakbari, Mehrzad Mehdizadeh, Amene Navaeian, Hamid Eshaghi, Bahareh Yaghmaei, Reihaneh Hosseinpour Sadeghi, Shiva Poormohammadi, Yasmine Mahmoudieh, Shima Mahmoudi

**Affiliations:** 1 Pediatric Infectious Diseases Research Center, Tehran University of Medical Sciences, Tehran, Iran; 2 Department of Infectious Diseases, Pediatrics Center of Excellence, Children’s Medical Center, Tehran University of Medical Sciences, Tehran, Iran; 3 Pediatrics Center of Excellence, Children’s Medical Center, Tehran University of Medical Sciences, Tehran, Iran; 4 Department of Radiology, School of Medicine, Tehran University of Medical Science, Tehran, Iran; 5 Division of Pediatric Intensive Care Unit, Pediatrics Center of Excellence, Children’s Medical Center, Tehran University of Medical Sciences, Tehran, Iran; 6 Department of Molecular and Cell Biology, University of California, Berkeley, USA; 7 Pediatric Infectious Disease Research Center, Children’s Medical Center Hospital, Tehran University of Medical Sciences, Dr. Gharib Street, Keshavarz Boulevard, Tehran, Iran

**Keywords:** COVID-19, SARS-CoV-2, MIS-C, Severity, Mortality

## Abstract

**Background:**

The rapid worldwide spread of severe acute respiratory syndrome coronavirus 2 (SARS-CoV-2) infections led to public health crises globally and the number of pediatric patients with Coronavirus Disease 2019 (COVID-19) is still rising. The aim of this study was to describe the epidemiological, clinical, laboratory, and imaging features of hospitalized patients with COVID-19 at an Iranian referral pediatrics hospital and to compare these parameters between hospitalized patients with and without severe disease, multisystem inflammatory syndrome in children (MIS-C) and children with acute COVID-19, as well as deceased and discharged cases.

**Methods:**

This study included hospitalized children and adolescents (≤ 18 years) with suspected COVID-19 who had positive results for SARS-CoV-2.

**Results:**

Among the 262 patients with suspected COVID-19, 142 confirmed COVID-19 cases were included in the study. A total of 11 children were diagnosed as MIS-C. The majority of the cases with MIS-C were male, (n = 9, 82%) which is significantly higher than children (n = 61, 47%) with acute COVID-19 (*P* = 0.03). Fifty patients (35%) were shown to have a more severe form of COVID-19. Ninety percent of the cases (n = 45) with severe COVID-19 had comorbidities that was significantly higher than cases with non-severe or mild disease (n = 41, 45%; *P* < 0.0001). A mortality rate of 10% was reported (n = 14). Ninety-three percent of the deceased cases (n = 13) had comorbidities that were significantly higher than discharged patients (n = 73, 57%; *P* = 0.009).

**Conclusion:**

The increasing number of children with severe COVID-19 is cause for great concern. Underlying diseases, mainly cardiovascular diseases, cancer, and malignancies, are associated with greater risk of development of severe COVID-19 and even death in children. On the other hand, pediatric patients with MIS-C usually develop a milder form of the disease. However, evaluation specific immunological responses in children to explore the delayed inflammatory syndrome are highly recommended.

## Background

The rapid worldwide spread of severe acute respiratory syndrome coronavirus 2 (SARS-CoV-2) infections led to public health crises globally and the number of pediatric patients with Coronavirus Disease 2019 (COVID-19) is still rising [1–7].

According to the previous reports, the prevalence of confirmed pediatric COVID-19 cases is around 1–2% of all diagnosed cases [8–10].

At the beginning of the COVID-19 outbreak, respiratory involvement was mainly observed in children, but in April 2020, a cluster of children with multi system inflammatory syndrome (MIS-C), a disorder with features similar to Kawasaki syndrome, emerged [11]. Because the manifestations of MIS-C patients appeared to overlap with acute COVID-19, less severe forms of disease and death were reported [12]. Therefore, defining the epidemiological and clinical features of the disease in large cohorts of pediatric patients is urgently needed. The aim of this study is to describe the epidemiological, clinical, laboratory, imaging features, and treatment of hospitalized patients with COVID-19 at an Iranian referral pediatrics hospital. These parameters were then compared between patients hospitalized with and without severe disease, MIS-C and children with acute COVID-19, and deceased and discharged cases.

## Methods

This study obtained ethical approval from Tehran University of Medical Sciences, Tehran, Iran (IR.TUMS.VCR.REC.1399.060). Assent was obtained from school-aged children, and written informed consent was provided by their parents/guardians prior to data collection.

This study was performed at the Children’s Medical Center, with a monthly turnover rate of more than 35,000 outpatients and 2500 inpatients.

The subjects of the study were hospitalized children with suspected COVID-19 who were hospitalized to Children’s Medical Centre hospital, an Iranian referral center for pediatric COVID-19.

Suspected cases of COVID-19 were defined in the presence of at least one of the following criteria: a history of contact with patients infected with COVID-19 or a history of contact with people with fever or respiratory symptoms within 14 days before onset.

All patients were symptomatic. Symptomatic cased were defined as those with fever, and/or respiratory signs, and/or diarrhea, and/or vomiting, and/or abdominal pain. MIS-C diagnosis was defined according to the following criteria: (1) history of close SARS-CoV-2 contact, (2) the presence of fever (> 38 °C) lasting for more than 24 h, (3) signs/symptoms of at least 2 organ involvement, (4) laboratory results displaying systemic inflammation [13].

Children younger than 18 years old with SARS-CoV-2 infection confirmed by real-time reverse transcription polymerase chain reaction (rRT*-*PCR) method using a nasopharyngeal swab, as previously described [13]. The rRT-PCR was performed according to the CDC protocol using the CDC 2019-Novel Coronavirus (2019-nCoV) rRT-PCR Diagnostic Panel. This includes N1 and N2 probes for detection of the virus nucleocapsid gene with RNase P (RP) used as an internal control. Inclusion criteria for MIS-C included positivity in real-time polymerase chain reaction (RT-PCR) positive or serology.

Between April 1st and August 13th, 2020, 287 children with suspected COVID-19 who were hospitalized to Children’s Medical Centre hospital and were tested for SARS-CoV-2. Twenty-five cases with MIS-C have been reported before and excluded from this study [13].

### Data collection

For patients who met eligibility criteria, the following groups of variables were collected: sex and age of the patients, presenting symptoms, duration of symptoms before presentation, comorbid conditions, disease severity, laboratory parameters, radiologic findings, administered therapies, and mortality.

Laboratory findings include leukocyte, neutrophil, and lymphocyte counts, hemoglobin, platelet count, oximetry saturation C-reactive protein, erythrocyte sedimentation rates, d-dimer, blood urea, serum creatinine, liver enzymes, fibrinogen, ferritin, lactate dehydrogenase, creatine phosphokinase, sodium, potassium, calcium, phosphorus, magnesium, albumin, procalcitonin, prothrombin time, partial thromboplastin time, and international normalized ratio. All testes were considered according to the laboratory reference values [1].

All symptoms reported at the time of presentation were documented, including COVID-19 symptoms. This includes fever (subjective or temperature ≥ 38 °C), cough, shortness of breath, abdominal pain, vomiting or diarrhea, tachypnea, myalgia, and rash.

The presence of household contacts was considered based on the presence of relevant symptoms related to COVOD-19 or laboratory confirmation of SARS-CoV-2.

A death due to COVID-19 was defined as a death resulting from a clinically compatible illness, in a confirmed COVID-19 case. There was no period of complete recovery from COVID-19 between illness and death; and no clear alternative cause of death that cannot be related to COVID disease should be found.

Patients with COVID-19 are considered to have severe illness if they have SpO_2_ < 93% (< 90% in premature infants), PaO_2_ < 60 mmHg, PaCO_2_ > 50 mmHg, a respiratory rate ≥ 70/min (≤ 1 year) and ≥ 50/min (> 1 year), or lung infiltrates > 50%; or other manifestations [14].

The CT imaging features of COVID-19 were reported as follows: ground-glass opacities and consolidations, opacity distribution, pleural effusion, fibrosis, and nodules. Moreover, bilateral lung involvement and number of affected lobes were considered. The chest radiographic findings were reported based on the density (ground-glass and consolidations), distribution, and lung involvements.

### Statistical analysis

All statistical analyses were performed using SPSS (Statistical Package for the Social Sciences) version 13.0 software (SPSS Inc). After an assessment of the normality of data using graphical and numerical (including statistical tests), normally distributed continuous variables were presented as means with standard deviations (SD). Otherwise medians (IQR: interquartile range) were used to compare the groups, using nonparametric methods.

A bivariate analysis was performed comparing laboratory parameters between patients with and without severe disease; MIS-C and other clinical presentations associated with COVID-19; and deceased and discharged cases. This was conducted using nonparametric tests (Mann–Whitney) or parametric tests (non-paired Student *t* test) for continuous variables as appropriate. Fisher exact tests or χ^2^ tests were used to compare categorical variables between different groups. A *P* < 0.05 was predetermined as the level of significance.

## Results

Among the 262 patients, 142 confirmed COVID-19 cases were included in the study. A total of 11 children were diagnosed as MIS-C (3 had positive SARS-CoV-2 rRT-PCR test and 8 had positive IgG anti-body against SARS-CoV-2) and the remaining 131 patients had other clinical presentations associated with COVID-19 with positive SARS-CoV-2 rRT-PCR tests.

The demographic and clinical characteristics of pediatric patients hospitalized with COVID-19 are shown in Table 1.

**Table 1:** The demographic and clinical characteristics of pediatric patients hospitalized with COVID-19, Tehran, Iran, 2020

Parameter	Acute COVID-19 (n = 131)	MIS-C (n = 11)	Severe (n = 50)	Non-severe (n = 92)	Discharged (n = 128)	Died (n = 14)
Age in years, median (IQR)	4 (1.1–10)	8 (1.6–10)	3.5 (0.6–8.5)	6 (2–10)	5 (1.6–10)	4 (0.4–11.7)
Male, no. (%)	61 (47)	9 (82)*	29 (58)	41 (45)	63 (49)	7 (50)
Comorbid conditions, no. (%)	84 (64)	2 (18)*	44 (88)	42 (49)*	73 (57)	13 (93)*
Hospital stay, median (IQR)	7 (4–12)	5.5 (5–6.75)	12 (8–21)	5 (4–8)*	7 (4–11)	19.5 (11.7–30)*
ICU admission, no. (%)	40 (30.5)	1 (9)	36 (72)	5 (5)*	28 (22)	13 (93)*
Mechanical ventilation required, no. (%)	23 (18)	0	23 (46)	0*	12 (9)	11 (79)*
Severe disease, no. (%)	50 (38)	0	–	–	36 (28)	14 (100)*
Known COVID + contact, no. (%)	49 (37)	7 (64)*	20 (40)	36 (39)	53 (41)	3 (21)
Symptoms						
Duration of symptoms in days prior to admission	2 (1–5)	4 (2–9)	3 (1–7)	2 (1–5)	2.5 (1–5)	1.5 (0.75–4.75)
Fever, no. (%)	100 (77)	11 (100)	36 (72)	75 (82)	102 (80)	9 (64)
Cough, no. (%)	51 (39.5)	3 (27)	23 (46)	31 (34)	48 (37.5)	6 (43)
Erythematous rash, no. (%)	9 (7)	8 (73)*	1 (2)	16 (17)*	7 (5)	0
Conjunctivitis, no. (%)	2 (1.5)	3 (27)*	1 (2)	4 (4)	5 (4)	0
Nausea/vomiting, no. (%)	41 (31.5)	3 (27)	13 (26)	31 (34)	41 (32)	3 (21)
Abdominal pain, no. (%)	27 (21)	4 (36)	6 (12)	25 (27)	30 (23)	1 (7)
Myalgia, no. (%)	25 (19)	3 (27)	8 (16)	20 (22)	25 (20)	3 (21)
Tachypnea no. (%)	56 (43)	3 (27)	35 (70)	24 (26)*	48 (37.5)	11 (79)*
Diarrhea, no. (%)	27 (21)	5 (45.5)*	6 (12)	26 (28)*	31 (24)	1 (7)
Shortness of breath, no. (%)	62 (47)	2 (20)	41 (82)	23 (25)*	51 (40)	13 (93)*
Treatment						
Oseltamivir, no. (%)	14 (11)	0	9 (18)	5 (5)*	11 (9)	3 (21)
Lopinavir/ritonavir, no. (%)	9 (7)	0	9 (18)	0*	4 (3)	5 (36)*
Hydroxychloroquine, no. (%)	65 (50)	1 (9)*	32 (64)	34 (37)*	53 (41)	13 (93)*
Vancomycin, no. (%)	36 (27.5)	1 (9)	26 (52)	11 (12)*	28 (22)	9 (64)*
Azitromycin, no. (%)	96 (73)	9 (82)	40 (80)	65 (71)	94 (73)	11 (79)
Cefotaxim, no. (%)	47 (36)	3 (27)	16 (32)	34 (37)	47 (37)	3 (21)
Steroids, no. (%)	20 (15)	9 (82)*	11 (22)	18 (20)	24 (19)	5 (36)
Mortality, no. (%)	14 (11)	0	14 (28)	0*	–	–

*ICU* intensive care unit

*P < 0.005

The median age of the children with MIS-C and children with acute COVID-19 was 8 years (IQR, 1.6–10 years) and 4 years (IQR, 1.1–10 years), respectively. The majority of the cases with MIS-C were male (n = 9, 82%). This was significantly higher than children with acute COVID-19 (n = 61, 47%; *P* = 0.03), while no significant differences between the sex and severity of disease and mortality were found (Table 1). Fifty-six patients (39%) had a documented adult family member or household contact with either symptom compatible with COVID-19 or a confirmed case of COVID-19 (Table 1). None of the patients had a history of international travel within 14 days before symptom onset.

Admission to pediatric critical care units was required for only 1 patient with MIS-C (9%), while it was required for 40 patients (30.5%; *P* = 0.177) with acute COVID-19. Mechanical ventilation was used for respiratory support in 23 patients (18%) with acute COVID-19 (Table 1).

Most patients were discharged (n = 128, 90%) with a median stay of 2 days (IQR, 1–5 days). Fifty patients (35%) showed signs of a severe form of COVID-19. Eighty-eight percent of the cases (n = 44) with severe COVID-19 had comorbidities that was significantly higher than cases with non-severe or mild disease (n = 42, 49%).

Among all patients, 86 (61%) had comorbidities of which cardiovascular (23, 27%) and cancer/malignancy (20, 23%) were the most common, followed by neurological disorders (6, 7%) and immunosuppressive conditions (5, 6%).

Most patients with MIS-C were previously healthy and only 2 (18%) had comorbidities (1 autism and 1 down syndrome). In contrast, 64% (n = 84) of the children with acute COVID-19 had comorbidities (*P* = 0.007). Among 142 patients, a mortality of 10% was reported (n = 14) (Table 1). Ninety-three percent of the cases (n = 13) who died had comorbidities (5 had cardiovascular diseases, 4 had cancer/malignancy, 3 had neurological disorders, an one case had cystic fibrosis) that were significantly higher than discharged patients (n = 73, 57%; *P* = 0.009).

### Clinical characteristics of patients

The clinical characteristics of patients with acute COVID-19 and MIS-C, severe and non-severe form of COVID-19, and deceased and surviving patients are shown in Table1.

Most patients had fever, and all patients with MIS-C presented with fever and variable combinations of erythematous rashes (n = 8, 73%), diarrhea (n = 5, 45.5%), abdominal pain (n = 4, 18%) (Table1). Conjunctival injection was noted in 3 patients (27%). This was more significantly seen in patients with MIS-C compared to the children with acute COVID-19 (*P* = 0.003). Moreover, there was a significant number of erythematous rashes seen in patients with MIS-C (73%) compared to the children with acute COVID-19 (7%) and patients with non-severe diseases compared to severe group (17% vs. 2%, respectively).

The median time to admission from development of symptoms in patients acute COVID-19 was 2 days (interquartile range [IQR], 1–5 days) but it was longer in patients with MIS-C (median, 4 days; IQR, 2–9 days); however, it was not significant (*P* = 0.28). Patients with severe COVID-19 were significantly more likely to present with respiratory distress compared with patients with in non-severe cases (82% vs. 25%). Diarrhea was more likely to present in patients with MIS-C (n = 5, 45.5%) compared to patients with acute COVID-19 (n = 27, 21%); however, it was not significant (*P* = 0.12) (Table 1).

### Laboratory investigations

Laboratory findings at admission in cases with acute COVID-19 and MIS-C, severe and non-severe form of COVID-19, and deceased and surviving patients are indicated in Tables 2 and 3. The level of lactate dehydrogenase was significantly higher in children with MIS-C compared to children with acute COVID-19 [671 U/L (IQR, 598–869 U/L) vs. 557 U/L (IQR, 408–727 U/L], respectively (*P* = 0.057) (Table 2).

**Table 2:** Laboratory findings of pediatric patients hospitalized with COVID-19, Tehran, Iran, 2020 on admission

Parameter	Acute COVID-19 (without MIS-C)	MIS-C	Non-severe	Severe	Discharged	Died
	Median (IQR)	Median (IQR)	Median (IQR)	Median (IQR)	Median (IQR)	Median (IQR)
Age (years)	4 (1.1–10)	8 (1.6–8)	6 (2–10)	3.5 (0.6–8.5)	5 (1.6–10)	4 (0.4–11.7)
White blood cell count (× 10^9^ cells per L)	8.6 (5.6–12.1)	6.6 (4.8–9.6)	7.6 (5.6–11.8)	9.6 (4.7–12.1)	8 (5.3–11.7)	9.3 (3.7–15.1)
Neutrophil count (× 10^9^ cells per L)	4.9 (2.5–8.4)	4.1 (2.6–7.5)	4.6 (2.5–7.6)	5.5 (2.4–9)	5 (2.5–8.2)	4.3 (2.8–8.9)
Lymphocyte count (× 10^9^ cells per L)	1.9 (1.1–3.3)	1.2 (0.7–2.3)	1.7 (1.1–3.4)	3 (2–3.3)	1.8 (1.1–3.1)	3.4 (0.5–5.2)
Haemoglobin (g/dL)	11.7 (9.7–13.1)	12.3 (10.7–13.2)	11.9 (10.7–13.2)	11.4 (9.3–12.9)	11.9 (10.2–13.2)	10.4 (8.4–12)
Platelet count (× 10^9^ cells per L)	268 (173–380)	258 (99–316)	273 (219–382)	231 (116–353)	274 (191–381)	148.5 (41–302)
Oximetry saturation (%)	90.4 (78–97)	90.3 (86.5–94.7)	89.6 (77.9–97.1)	92.2 (81.2–97.2)	90.4 (78–97)	90.9 (82–98)
Urea (mmol/L)	12 (8–15)	10 (8–17)	12 (9–15)	10 (7–15.5)	11 (1.1–3.1)	20 (11–26.2)
Creatinine (µmol/L)	0.6 (0.4–0.7)	0.5 (0.5–0.6)	0.6 (0.5–0.7)	0.5 (0.3–0.6)	0.5 (0.5–1.4)	0.5 (0.3–0.92)
Creatine phosphokinase (U/L)	61 (33–101)	69 52–279)	67.5 (37.5–100)	51 (31–115.5)	64 (36–101)	51 (30.5–371.5)
Lactate dehydrogenase (U/L)	557 (408–727)	671 (598–869)	527(383–663.5)	680(531.5–984)	550 (400–708)	1121 (700–1617)
Calcium (mg/dL)	8.9 (8.5–9.5)	8.5 (8.1–9.3)	9.1 (8.4–9.5)	8.7 (8.3–9.3)	8.9 (8.5–9.4)	8.6 (8.3–9.6)
Phosphorus (mg/dL)	4.2 (3.8–4.9)	4.1 (3.6–4.6)	4.1 (3.7–4.8)	4.5 (3.7–5.1)	4.2 (3.7–5.1)	4.3 (3.8–4.8)
Magnesium (mg/dL)	1.9 (1.7–2.1)	2.1 (1.9–2.4)	1.9 (1.7–2.1)	1.9 (1.7–2.2)	1.9 (1.8–2.1)	1.8 (1.6–2.4)
Sodium (meq/L)	135 (132–138)	134.5 (133–137)	136 (133–138)	134 (131–137.5)	136 (133–138)	133 (131–136.5)
Potassium (meq/L)	4.1 (3.9–4.5)	3.9 (3.8–4.5)	4.1 (3.9–4.5)	4.2 (3.9–4.6)	4.1 (3.9–4.5)	4.4 (4–4.7)
Prothrombin time (s)	13.3 (12.5–14)	12.8 (12.5–14)	13.2 (12.5–14)	13.7 (12.5–14.7)	13.3 (12.5–14)	14.6 (13.3–19.2)
Partial thromboplastin time (s)	33 (30–38)	34 (33–40)	33 (30–37)	34 (31–41.5)	33 (30–37.7)	35 (31–41.5)
International normalized ratio (mm/h)	1.1 (1–1.2)	1 (1–1.2)	1.1 (1–1.2)	1 (1.1–1.3)	1.1 (1–1.2)	1.1 (1.1–1.97)
C-reactive protein (mg/L)	18 (5–44.5)	19 (4–54)	17.5 (4–42.5)	19.5 (6.7–49.2)	13 (4–42)	43 (24.5–109)
Erythrocyte sedimentation rate	24 (12–44.7)	24 (12–46)	21 (12–44)	27 (10.2–51.5)	23 (12–44)	26 (3–90)
Alanine aminotransferase (U/L)	32 (22–39.5)	35 (27–124)	26.5 (21.2–37.7)	34.5 (29–60.5)	30 (22–40)	34 (21.5–205.5)
Aspartate aminotransferase (U/L)	21 (13–41.5)	23 (14–78)	20.5 (13–39)	24.5 (15.2–72.7)	22 (13–40)	29 (16–80)
Procalcitonin (ng/mL)	0.02 (0.01–0.3)	0.1 (0.02–0.5)	0.02 (0.01–0.27)	0.02 (0.01–0.77)	0.02 (0.01–0.27)	2 (0.02–10)
Albumin (g/dL)	4 (3.4–4.5)	4.4 (4.1–4.4)	4.3 (3.9–4.5)	3.7 (3.1–4.3)	4.2 (3.6–4.5)	3.6 (2.7–4)
Fibrinogen (mg/dL)	323 (203–490)	411 (225–606)	427 (225.5–534.5)	296 (202.5–490)	395.5 (244–498)	296 (80–605)
Ferritin (ng/mL)	193 (101–546)	135 (91–353)	147 (91–359)	460 (131–1310)	188 101–512)	1890 (76–2490)
d-Dimer (pg/mL)	0.9 (0.38–6)	1.8 (1.7–1.8)	3.95 (1.7–264)	0.7 (0.3–3.1)	1.7 (0.6–4.5)	1.8 (0.2–7785)

**Table 3:** Laboratory findings of pediatric patients hospitalized with COVID-19, Tehran, Iran, 2020 on admission among different groups

Parameter	All cases	Non-severe	Severe	Discharged	Died	Acute COVID-19	MIS-C
Decreased WBC count	21 (15) [n = 139]	14 (16) [n = 90]	7 (14) [n = 49]	18 (14) [n = 125]	3 (21) [n = 14]	19 (15) [n = 128]	2 (18) [n = 11]
Normal WBC count	66 (48) [n = 139]	46 (51) [n = 90]	20 (41) [n = 49]	61 (49) [n = 125]	5 (36) [n = 14]	59 (46) [n = 128]	7 (64) [n = 11]
Increased WBC count	52 (37) [n = 139]	30 (33) [n = 90]	22 (45) [n = 49]	46 (37) [n = 125]	6 (43) [n = 14]	50 (39) [n = 128]	2 (18) [n = 11]
Decreased neutrophil count	57 (42) [n = 136]	39 (44) [n = 89]	18 (38) [n = 47]	52 (42) [n = 124]	5 (42) [n = 12]	52 (42) [n = 125]	5 (46) [n = 11]
Normal neutrophil count	53 (39) [n = 136]	32 (36) [n = 89]	21 (45) [n = 47]	48 (39) [n = 124]	5 (42) [n = 12]	49 (39) [n = 125]	4 (36) [n = 11]
Increased neutrophil count	26 (19) [n = 136]	18 (20) [n = 89]	8 (17) [n = 47]	24 (19) [n = 124]	2 (16) [n = 12]	24 (19) [n = 125]	2 (18) [n = 11]
Decreased lymphocyte count	21 (15) [n = 136]	14 (16) [n = 89]	7 (15) [n = 47]	18 (14) [n = 124]	3 (25) [n = 12]	18 (14) [n = 125]	3 (27) [n = 11]
Normal lymphocyte count	93 (68) [n = 136]	61 (68) [n = 89]	32 (68) [n = 47]	90 (73) [n = 124]	3 (25) [n = 12]	85 (68) [n = 125]	8 (73) [n = 11]
Increased lymphocyte count	22 (16) [n = 136]	14 (16) [n = 89]	8 (17) [n = 47]	16 (13) [n = 124]	6 (50) [n = 12]	22 (18) [n = 125]	0
Decreased platelet count	30 (21) [n = 140]	14 (16) [n = 91]	16 (33) [n = 49]	23 (18) [n = 126]	7 (50) [n = 14]	26 (20) [n = 129]	4 (36) [n = 11]
Normal platelet count	92 (66) [n = 140]	65 (71) [n = 91]	27 (55) [n = 49]	86 (68) [n = 126]	6 (43) [n = 14]	85 (66) [n = 129]	7 (64) [n = 11]
Increased platelet count	18 (13) [n = 140]	12 (13) [n = 91]	6 (12) [n = 49]	17 (14) [n = 126]	1 (7) [n = 14]	18 (14) [n = 129]	0
Elevated urea	15 (11) [n = 137]	6 (7) [n = 88]	9 (18) [n = 49]	8 (6.5) [n = 123]	7 (50) [n = 14]	15 (12) [n = 126]	0
Elevated creatine phosphokinase	11 (11) [n = 98]	6 (9) [n = 68]	5 (17) [n = 30]	9 (10) [n = 90]	2 (25) [n = 8]	8 (9) [n = 89]	3 (33) [n = 9]
Elevated lactate dehydrogenase	30 (26) [n = 117]	15 (19) [n = 80]	15 (40.5) [n = 37]	23 (21.5) [n = 107]	7 (70) [n = 10]	25 (24) [n = 106]	5 (45.5) [n = 11]
Elevated alanine aminotransferase	53 (51) [n = 104]	29 (43) [n = 68]	24 (67) [n = 36]	45 (49.5) [n = 91]	8 (61.5) [n = 13]	47 (50.5) [n = 93]	6 (54.5) [n = 11]
Elevated aspartate aminotransferase	35 (36) [n = 97]	21 (33) [n = 63]	14 (41) [n = 34]	29 (34.5) [n = 84]	6 (46) [n = 13]	29 (34) [n = 86]	6 (54.5) [n = 11]
Elevated C-reactive protein	88 (69) [n = 128]	53 (65) [n = 82]	35 (76) [n = 46]	73 (65) [n = 115]	13 (100) [n = 13]	81 (69) [n = 117]	7 (64) [n = 11]
Elevated Erythrocyte sedimentation rate	102 (79) [n = 129]	69 (81) [n = 85]	33 (75) [n = 44]	93 (80) [n = 116]	9 (69) [n = 13]	92 (78) [n = 118]	10 (91) [n = 11]
Elevated prothrombin time	52 (40) [n = 129]	27 (32) [n = 84]	25 (56) [n = 45]	43 (37) [n = 116]	9 (69) [n = 13]	49 (41.5) [n = 118]	3 (27) [n = 11]
Elevated procalcitonin	31 (33) [n = 94]	22 (34) [n = 64]	9 (30) [n = 30]	28 (32) [n = 88]	3 (50) [n = 6]	25 (30) [n = 83]	6 (54.5) [n = 11]
Decreased albumin	50 (73) [n = 68]	6 (15) [n = 40]	12 (43) [n = 28]	14 (24) [n = 58]	6 (60) [n = 10]	17 (29) [n = 59]	1 (11) [n = 9]

*WBC* white blood cells

The proportion of normal white blood cells in COVID-19 patients in children with MIS-C was 64%, and it was 46% in children with acute COVID-19. Leukocytosis was observed in 18% and 39% of children with MIS-C and children with acute COVID-19, respectively. Eighteen percent of patients with MIS-C had leukopenia. It was seen in 15% of other cases. The higher proportion of patients with high procalcitonin, creatine phosphokinase, lactate dehydrogenase, aspartate aminotransferase, erythrocyte sedimentation rate was seen in patients with MIS-C compared to children with acute COVID-19 (54.5% vs. 30%, 33% vs. 9%, 45.5% vs. 24%, 54.5% vs. 34%, and 91% vs. 78%, respectively); however, these differences were not significant (Table 3).

### Laboratory findings at admission in patients with non-severe and severe disease

On admission, the white blood cell counts were generally normal (median,7.6 × 10^3^/μL; IQR, 5.6–11.8 × 10^3^/μL in patients with non-severe disease and median, 9.6 × 10^3^/μL; IQR, 4.7–12.1 × 10^3^/μL in patients with severe disease). Lymphopenia was seen in 14 patients with non-severe disease (16%) and 7 patients with severe disease (15%), respectively. Absolute neutrophil counts at admission did not differ significantly in patients with and without severe disease (median 4.6 × 10^3^/μL vs 5.5 × 10^3^/μL, respectively). Thrombocytopenia was seen in 16% (n = 14) and 33% (n = 16) of patients with and without severe disease, respectively (*P* = 0.057) (Table 3).

Lactate dehydrogenase was significantly elevated in patients with severe disease [680 (U/L); IQR, 531.5–984 U/L vs. 527 U/L; IQR, 383–663.5 U/L; *P* = 0.017]. Fifteen out of 37 cases (40.5%) with severe COVID-19 had an increased level of lactate dehydrogenase. Alanine aminotransferase was significantly elevated at admission for patients with severe disease compared to patients with mild disease [34.5 (U/L); IQR, 29–60.5 U/L vs. 26.5 U/L; IQR, 21.2–37.7 U/L; *P* = 0.011].

### Laboratory findings at admission in deceased and surviving patients

Normal white blood cell counts were seen in 73% of patients who were discharged, but in only 25% of the patients who are now deceased. Lymphopenia was seen in 25% of discharged patients and 14% of the patients who are now deceased, respectively. Lymphocytosis was found in half of the deceased patients, while it was observed in only 13% of discharged cases (*P* = 0.001). Thrombocytopenia was seen in 50% (n = 7) and 18% (n = 23) of deceased and discharged patients, respectively (*P* = 0.023) (Table 3).

According to laboratory analysis, urea was significantly elevated in deceased patients compared to the discharged patients [20 (mmol/L); IQR, 11–26.2 mmol/L vs. 11 mmol/L; IQR, 1.1–3.1 mmol/L; *P* = 0.004]. Lactate dehydrogenase was significantly elevated at admission for deceased patients compared to the discharged patients [1121 (U/L); IQR, 700–1617.5 U/L vs. 550 U/L; IQR, 400–708 U/L; *P* < 0.001]. The level of procalcitonin [2 ng/mL (IQR, 0.02–10 ng/mL) vs. 0.02 ng/mL (IQR, 0.01–0.27 ng/mL); *P* = 0.387], prothrombin time [14.6 s (IQR, 13.3–19.2 s) vs. 13.3 s (IQR, 12.5–14 s); *P* = 0.019], and C-reactive protein [43 mg/L (IQR, 24.5–109 mg/L) vs. 13 mg/L (IQR, 4–42 mg/L); *P* = 0.017] was higher in children who are deceased compared to those who were discharged. The level of albumin was significantly low in patients who are now deceased compared to the discharged patients (3.6 g/dL; IQR, 2.7–4 g/dL vs. 4.2 g/dL; IQR, 3.6–4.5 g/dL; *P* = 0.036) (Table 2).

### Radiographic findings

Imaging features for patients are summarized in Table 4. According to chest x-ray and computerized tomography (CT), 48% and 24% had normal results, respectively. Chest radiographs were performed for 60 patients (42%). Findings included bilateral patchy or ground glass opacities (n = 23, 38%) and consolidation (n = 13, 22%). The most common pattern on chest CT was ground-glass opacity (n = 35, 59%). Consolidation and bilateral distribution were seen in 28 (47%) and 31 (53%) cases, respectively; and 22 (37%) had involvement of two or more lobes found in the chest CT.

**Table 4:** Findings at initial chest CT and radiographic examination of pediatric patients hospitalized with COVID-19, Tehran, Iran, 2020

Findings	N	%
CT scan (n = 59)		
Normal	14	24
Ground-glass opacities	35	59
Consolidation	28	47
Number of lobe involvement		
0	2	3
1	9	15
2	9	15
3	7	12
4	4	7
6	2	3
Diffuse	12	20
Bilateral lung disease	31	53
Pleural effusion	6	10
Nodule	13	22
Fibrosis	7	12
Opacity distribution	41	69
Chest radiography (n = 60)		
Normal	29	48
Consolidation	13	22
Ground-glass opacities	23	38
Central distribution	15	25
Peripheral	21	35
Diffuse distribution	12	20
Lobe involvement		
0	6	10
1	6	10
2	7	12
3	2	3
4	2	3
Diffuse	8	13

Abnormal chest x-ray and CT findings were found in 77% (23 out of 30) and 60% (27 out of 45) patients with severe disease that was significantly higher than patients without severe disease [8 out of 30 (27%); *P* < 0.0001 and 1 out of 14 (7%); *P* = 0.001], respectively. Moreover, abnormal chest x-ray and CT findings were found in 83% and 100% of deceased patients (10 out of 12; and 11 out of 11) compared to the discharged patients (42%, 20 out of 48; and 71%, 34 out of 48).Among 53 patients who underwent both chest x-ray and CT scan, 11 (21%) and 28 patients (53%) showed normal and abnormal findings with both tests, respectively. Thirteen patients (25%) with normal chest x-ray showed abnormal finding in CT scan.

### Administered therapy

Hydroxychloroquine was administered to 66 patients (46%) of children mainly with acute COVID-19 than MIS-C. Azithromycin was administered in the majority of cases (n = 105, 74%). Eighty-two percent of patients with MIS-C depends on the constellation of clinical findings as well as 15% of children with acute COVID-19 with severe manifestations were treated initially with glucocorticoids. Glucocorticoid therapy with methylprednisolone was initially given at a dose of 2 mg/kg/day in two divided doses. Oseltamivir, lopinavir/ritonavir, hydroxychloroquine, and vancomycin were administered significantly higher in cases with severe diseases (Table 1).

## Discussion

Although there are relatively extensive reports available for adult COVID-19 patients, our knowledge about the epidemiological and clinical characteristics, laboratory findings and mortality of pediatric COVID-19 is quite limited. To our knowledge, this is the largest study in Iran that has reported the epidemiological, clinical, laboratory, radiological characteristics, treatment and outcomes of the pediatric COVID-19 patients. All data was compared between patients with MIS-C and children with acute COVID-19, severe and non-severe disease, and deceased and discharged cases.

During the early stages of the COVID-19 outbreak, children mainly with respiratory presentations associated with COVID-19 were hospitalized, but the situation changed rapidly. Recently, the number of hospitalized patients with MIS-C is increasing. During a period of more than 6 months, a total of 322 COVID-19 suspected cases were hospitalized in our hospital and tested for SARS-CoV-2. In our previous study from March 7th to March 30th of 2020, it was reported that a total of 35 COVID-19 suspected cases were hospitalized in our hospital, and 11 of them had positive rRT-PCR results [14]. Moreover, we reported 25 cases with MIS-C between March 7th and June 23th of 2020 [13]. With the exclusion of the 36 confirmed cases from this study, we described 142 confirmed cases with COVID-19. Therefore, among COVID-19 suspected cases, the prevalence of confirmed COVID-19 in our hospital during the period of more than 6 months was 55% (n = 178).

In our study, fever was the main clinical manifestation of COVID-19. Although cough is considered one of the most common symptoms in children [9, 15–17], it was noted in 27% and 39.5% of children with MIS-C and with other clinical presentations associated with COVID-19, respectively. However, in our previous report written at the initiation of the epidemic, it was reported in 80% of the cases [13]. Although myalgia is not usually reported in children [8] it was reported in 27% of children with MIS-C and 19% of children with other clinical presentations associated with COVID-19.

The majority of children with multisystem inflammatory syndrome have serologic evidence of infection, but approximately a third (n = 3) tested positive for SARS-CoV-2 by rRT-PCR. That is inconsistent with our previous study [13] and other reports [18–20]. It is possible that the virus had already been cleared from the upper respiratory tract of these patients, and they already had IgG-type antibodies [21].

In this study, we found that pediatric patients with underlying diseases could be at increased risk for greater severity and a worse outcome of the disease [22]. Ninety-three percent of the deceased cases (n = 13) had comorbidities. This is significantly higher than discharged patients (n = 73, 57%; *P* = 0.009). Cardiovascular disease, cancer and malignancies were the most common underlying diseases in our study. Although the mechanism of probable association between cardiovascular disease and COVID-19 is not clear, it might be due to the presence of angiotensin-converting enzyme 2 (ACE2) receptors on cardiac muscle cells [22]. On the other hand, patients suffering from cancer or malignancy are at a higher risk of developing a more severe form of COVID-19, most likely due to their weakened immune response [22]. However, there is limited data in this regard for children.

Typical COVID-19 laboratory markers, such as lymphocytopenia, the most common sign of a low blood count in adults [23], are uncommon in children. Leukocyte counts in children are often normal; however, it was normal in only 25% of patients who are deceased. Lymphocytosis was seen in 50% of later deceased children with COVID-19. This was significantly higher than in discharged cases. It might, therefore, be related to poorer prognosis in children; however, their oncological underlying diseases should not be ignored.

The overproduction of proinflammatory cytokines plays a major role in the pathogenesis of COVID-19, leading to an increased risk of multi-organ failure [24]. Similar to previous studies, evidence of infection or inflammation including elevated concentrations of C-reactive protein, procalcitonin, ferritin, and d-dimers was reported [25, 26]. The median level of C-reactive protein was significantly higher in children with MIS-C; however, the proportion of patients with elevated C-reactive protein was not significantly different between children with MIS-C and with acute COVID-19.

That was significantly higher than cases with non-severe COVID-19 (15 out of 80, 19%; *P* = 0.022). Liver enzymes, particularly alanine aminotransferase, were significantly elevated in cases with severe COVID-19. In previous studies, they were frequently normal in pediatric patients, maybe due to the evaluation of these enzymes in asymptomatic or non-severe children with COVID-19 [8]. However, abnormal liver function was reported in half of the 50% of the severe and critically ill patients [27].

In contrast to adults where abnormalities in chest CT images might be detected in the majority of patients [23], it was found in 76% of tested patients. CT scans, however, showed more extensive lung involvement than the X-ray. No abnormalities in radiographs were found in 48% of cases. This is consistent with previous reports [8]. Patchy consolidation and ground glass opacities were the most common radiographic features that are in consistent with previous reports [8, 13, 28].

At first, administration of hydroxychloroquine and azithromycin for treatment of COVID-19 patients was recommended [29]. It has been reported that hydroxychloroquine treatment might lead to reduction/disappearance of viral load in COVID-19 patients, and its effect is reinforced by azithromycin [30, 31]. However, we cannot definitively rule out either a substantial benefit or harm of using hydroxychloroquine.

Most children with severe manifestations are treated with both intravenous immune globulin (IVIG) and glucocorticoids. However, due to equal or even better efficacy, as well as the lower price, steroids are used as a better choice than IVIG in our country [13]. In our study, patients with MIS-C did not receive IVIG and there were no deaths in this group of patients.

In our study, the mortality rate was 10% and 14 out of 142 patients died. It should be noted that in our study, comorbidities were higher than previous reports in children [32]. Therefore, the key issue regarding the mortality rate could be due to the presence of comorbidities in children.

To our knowledge, the sample size of this study was relatively large. However, it has some limitations. First, there are some limitations regarding nucleic acid tests, including high rates of false negative results due to the late collection of the sample, inadequate or insufficient viral material in the specimen, or laboratory error. Second, several children with complicated diseases are referred to our hospital. Therefore, our study may represent a more severe form of COVID-19, and the results should be interpreted with caution. Third, some patients are still hospitalized, and the long-term outcome and sequelae may need further follow-up.

## Conclusion

In conclusion, the increasing number of children with severe COVID-19 is cause for great concern. Underlying diseases, mainly cardiovascular diseases, cancer and malignancies, are associated with a greater risk of patients developing a more severe form of COVID-19, and even death. On the other hand, pediatric patients with MIS-C usually develop a milder form of the disease. However, evaluation of specific immunological responses in children to explore the delayed inflammatory syndrome is highly recommended.

## Data Availability

The patient data and datasets used and/or analyzed during the current study are available from the corresponding author on reasonable request.

## Abbreviations

SARS-CoV-2: Severe acute respiratory syndrome coronavirus 2
COVID-19: Coronavirus disease 2019
MIS-C: Multi system inflammatory syndrome
rRT-PCR: Real-time reverse transcription polymerase chain reaction
ACE2: Angiotensin-converting enzyme 2
ICU: Intensive care unit
IQR: Interquartile range
CT: Computerized tomography
WBC: White blood cells
